# The Barriers and Enablers of Primary Healthcare Service Transition From Government to Community Control in Yarrabah: A Grounded Theory Study

**DOI:** 10.3389/fpubh.2021.616742

**Published:** 2021-10-14

**Authors:** Janya McCalman, Crystal Sky Jongen, Sandy Campbell, Ruth Fagan, Kingsley Pearson, Suzanne Andrews

**Affiliations:** ^1^Centre for Indigenous Health Equity Research, Central Queensland University, Cairns, QLD, Australia; ^2^Molly Wardaguga Research Centre, Charles Darwin University, Darwin, NT, Australia; ^3^Gurriny Yealamucka Health Service, Yarrabah, QLD, Australia

**Keywords:** community control, self-determination, governance, transition, Indigenous

## Abstract

**Introduction:** Consistent with the aspirations of First Nations Australians for community control of healthcare services, 123/196 (63%) of Australia's First Nations-specific primary health care services are community-controlled. Yet despite policy commitment over 30 years, the transition of government-run First Nations' primary healthcare services to First Nations community control has been slow. This paper identifies the barriers and enablers to transitioning the delivery of primary healthcare services from Queensland Health to Gurriny Yealamucka community-controlled health service in Yarrabah.

**Methods:** Grounded theory methods were used to select 14 Gurriny and Queensland Health (QH) personnel involved in the transition for interview and to analyse these interview transcripts and 88 Gurriny organisational documents.

**Results:** Barriers and enablers to transition were identified at three levels: those internal factors within Gurriny, external factors directly related to the government handover, and broader structural and policy factors outside the control of either Gurriny or QH. Barriers at the Gurriny organisational level were an internal lack of experience and capacity, and varying levels of community confidence; enablers were leadership stability and capacity, community mandate, relationships with partner organisations, and ability to provide service continuity. Barriers in Gurriny's relationship with QH were a lack of certainty, transparency and prioritisation of the transition process; systemic racism; difficulties obtaining and maintaining the necessary workforce; limited resources including insufficient, unstable and inappropriate funding support; and problems with information sharing; enablers were performance frameworks to keep transition progress on track. Barriers in broad policy environment were an unsupportive Queensland government policy environment; government bureaucracy; and delays, conflicts and divisions; enablers were high-level government support and commitment.

**Conclusions:** The evaluation of Yarrabah's transition process suggests that future such transitions will require planning and commitment to a long-term, multi-faceted and complex process, encompassing the required level of authorisation and resourcing. This case example of a transition from government to community control of PHC highlighted the ongoing power issues that are faced every day by community-controlled organisations that co-exist with mainstream health systems within a colonial power structure.

## Introduction

First Nations peoples globally value their right to “retain their Indigenous values and traditions, ways of life and their languages and cultures, and to do so in a contemporary context” [(1), p. 156]. They have sought this autonomy despite government “policies of dispossession, marginalisation, assimilation and integration,” and related experiences of discrimination, pre-judice and indifference [(2), p. 10]. As clearly cited in the Uluru Statement from the Heart by Aboriginal and Torres Strait Islander (hereafter respectfully termed First Nations) Australians, “Our Aboriginal and Torres Strait Islander tribes were the first sovereign Nations of the Australian continent … and possessed it under our own laws and customs…. This sovereignty is a spiritual notion*.…* With substantive … structural reform, we believe this ancient sovereignty can shine through” [(3), p. iv]. Only with self-determination will First Nations peoples be able to fully overcome the legacy of Australia's colonisation and dispossession including current disadvantage (4, 5).

Self-determination (or community control) of an organisation is achieved when it attains real power to make decisions through community boards and management, such as how to utilise resources (6). The first Aboriginal Community-Controlled Healthcare Organisation (ACCHO) was established in 1971 in the context of ongoing resistance by First Nations peoples to widespread systemic racism, ongoing processes of colonisation and dispossession (6–8), and a dearth of government support and funding (9, 10). In Australia, 123/196 (63%) of First Nations primary healthcare services are currently community controlled, with the remaining 63 (32%) being government-run and 12 (6%) non-government operated (11).

Most of the current ACCHOs were established as community controlled from the start; a minority were transitioned from previously government-run services. Transition requires the devolution of power and authority by the state or territory government over First Nations' core institutions, goals and identity, as well as strengthening of the capacity of a First Nations community controlled organisation to renegotiate bureaucratic, legal and policy arrangements with the state (2). This research paper examines the barriers and enablers encountered throughout one attempt to attain self-determination through the first transition of a government-run primary healthcare (PHC) service to First Nations community control in Queensland. The healthcare service transition was negotiated in Australia's largest discrete First Nations community and one of its most disadvantaged—Yarrabah.

## Background

As well as through the 196 First Nations PHCs across Australia, PHC services are available to First Nations Australians through mainstream services such as the Commonwealth government subsidised, privately owned general practise PHCs, or state funded and provided hospitals (12). However, barriers have been documented relating to the accessibility, affordability, cultural acceptability and appropriateness of mainstream PHC to First Nations peoples' health needs (12–15). For example, a recent systematic review found that “Aboriginal people fare worse than non-Aboriginal people when accessing usual (mainstream) healthcare services” [(16), p. 314]; with mainstream health services and standard, non-tailored care not being responsive to community health needs (17). In Queensland, a report by the Anti-Discrimination Commission and Aboriginal and Islander Health Council found that government-run mainstream hospital and healthcare services were “not taking [their] responsibilities to Close the Indigenous Health Gap seriously” [(18), p. 14], and identified the structural conditions for institutional (or systemic) racism. Systemic racism occurs when in-built discrimination “systematically reflect[s] and produce[s] racial inequalities…” [(19), p. 438]. Such barriers result in later presentation at PHC services and at hospitals with more advanced and complex health issues than those of non-Aboriginal Australians, thereby contributing to an increased burden of disease and reduced quality of life (13, 20).

The ACCHO sector provides an important expression of the principle of self determination as “a proven mechanism for Aboriginal people to take responsibility over their own health matters” (6, 9, 21). ACCHOs are incorporated organisations initiated and governed by First Nations community members. They deliver holistic and culturally appropriate health services to the community (22). They are funded by both state and Commonwealth governments, using multiple funding models. ACCHOs address many of the healthcare access barriers because services and programs are grounded in local values and culture (23), adopt the First Nations concept of holistic health that encompasses social, political and cultural determinants of health (8, 9, 21), and are accountable to the interests, needs, values, vision and concerns of community members (2, 21, 23). They address affordability barriers by providing free primary healthcare, and accessibility barriers by providing transport, outreach and childcare support services (23). Non-Indigenous people are also cared for in these clinics, but First Nations people represent 82% of all clients (11). Through their cultural-centredness, and comprehensive and flexible approach to primary healthcare, ACCHOs are similar to Indigenous health services internationally (24).

The transition of state or territory run PHC services to First Nations community control is a complex process (25). For the past 30 years, Commonwealth and state governments in Australia have provided a funding and policy commitment to community control (26). For example, the National Aboriginal and Torres Strait Islander Health Plan 2013–2023 promotes a “robust, strong, vibrant and effective community controlled health sector” in which “individuals and community actively engage in decision making and control” [(27), p. 7]. Since the mid-late 90's this trend has included the support of the Queensland and Northern Territory health authorities for transferring PHC services delivered in First Nations communities to community control (25, 26). But despite policy commitment and significant investments in health reforms, there have been few successful transitions (28). For example, Northern Territory reforms to promote community control over PHC governance and service delivery produced the transfer of only one clinic during the period 2011–14, and no further proposals being accepted by the government (8).

Past evaluations of documented examples, particularly in regional and remote Northern Territory, Queensland and South Australian communities, have ascertained a range of enablers to successful transitions. These included a recognition by governments that their dominant governance arrangements required institutional change, including: the presence of niche alternative practises within government departments that provide a template for change; effective authorisation and sustained commitment through a continuity of leadership from ministers and senior government officials; and explicit measures to address systemic racism (8). As well, transitions required adequate time, funding and capacity (5, 26, 28). However, many more evaluations document the barriers to transition efforts (28). For example, barriers to transitioning the regional ACCHOs, Miwatj Health Aboriginal Corporation in the Northern Territory and Apunipima Cape York Health Council in Queensland included: poor coordination and role clarity between state/territory and ACCHO providers and between funding agencies and ACCHOs; short-term funding contracts; challenges associated with regionalising governance; accountability for effective care, access and responsiveness to communities; two-way accountability with funders; and a need for increased funding to cover rural/remote costs and improve needs-based equity (16). These barriers limited the success of transition to community controlled governance of only one each of their several regional clinics (the Yirrkala and Mossman clinics, respectively) (28). Elucidating these barriers and enablers allows government and community stakeholders to streamline processes and avoid the repetition of costly and damaging practises that hinder such efforts (29).

To that end, this paper presents the results of the recent evaluation of the successful transition to community control of PHC in Yarrabah from Queensland Health to Gurriny Yealamucka Health Service (hereafter Gurriny) (30). It documents the enablers and barriers to the transition process so that other communities aspiring to transition, and government partners wanting to support them, can improve future transitions. The research question was: What were the enablers that supported the transition of the delivery of PHC services to First Nations community control in Yarrabah, and what were the barriers to this transition?

## Methods

### Research Approach

We applied the Indigenous research and data sovereignty principles of ownership, control, access and possession (OCAP®) within the research (31). The research was contracted to Gurriny by Queensland Health (QH). “Ownership” was enacted through Gurriny control of the research funding, governance and research partnership with Central Queensland University (CQU)'s Centre for Indigenous Health Equity Research (CIHER) through a Research Services Agreement, and their oversight throughout. “Control” was asserted through a steering committee established to guide the research, that was chaired and coordinated by Gurriny, and included representatives from the Aboriginal and Torres Strait Islander branch of QH, Cairns and Hinterland Hospital and Health Service (CHHHS), Queensland Aboriginal and Islander Health Council (QAIHC), Gurriny, and the CIHER research team. Seven of the nine members were First Nations people. Data from participant interviews were secured on a CQUniversity data management server but “access” to the aggregated findings was provided by CIHER researchers to Gurriny staff through plain English reports and presentations of the findings. “Possession” was enabled through Gurriny ownership of the final report and co-authorship of this paper (31). Further details of the approach and methods are provided in a companion paper on the processes and strategies of transition (25).

### The Provision of Primary Healthcare to the Yarrabah Community

**Figure 1:**
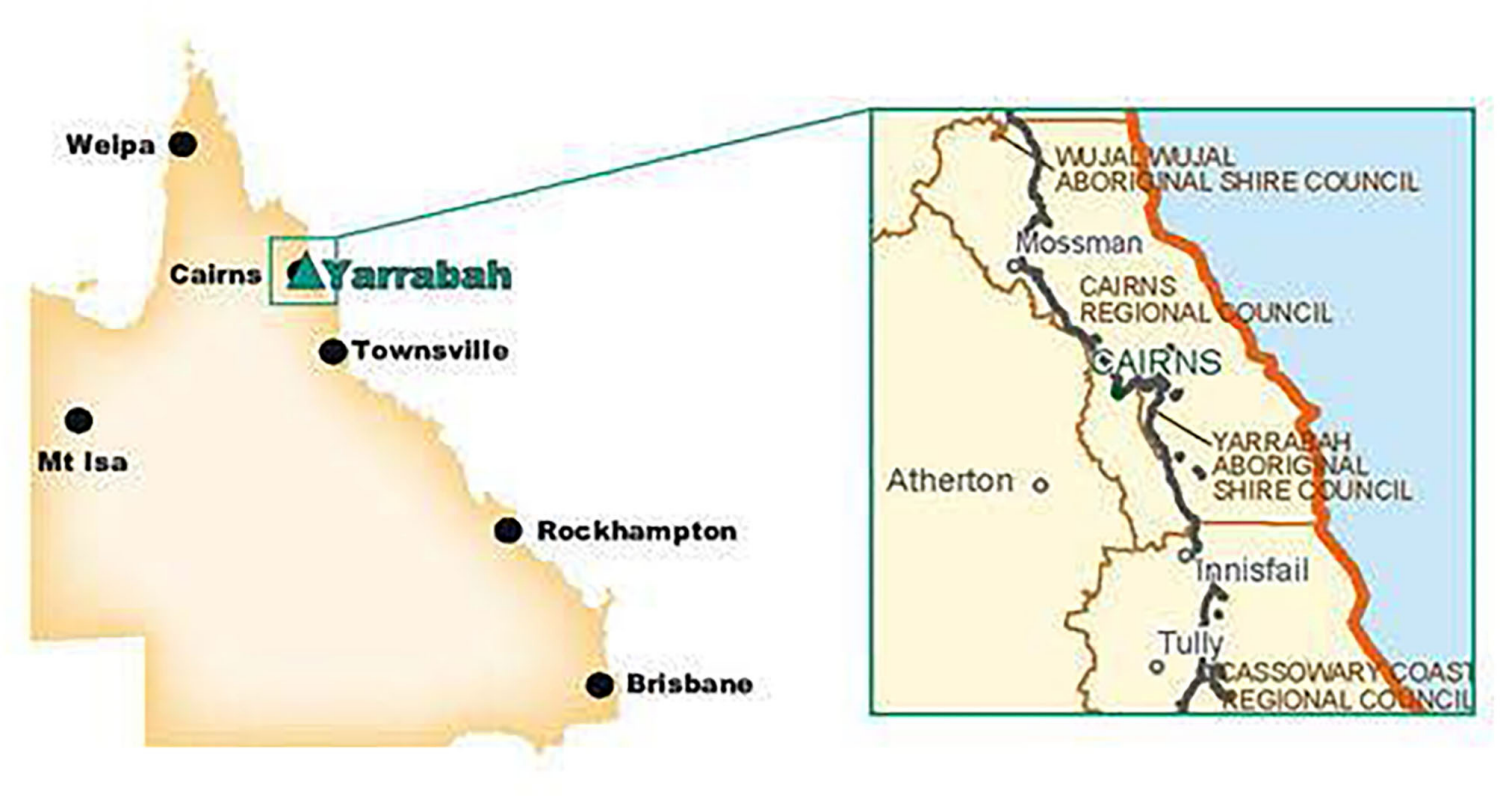
Yarrabah. Source: Bentleys (32).

Yarrabah is a discrete First Nations community in Far North Queensland, 52 km south east of Cairns (Figure 1). In 1892, an Anglican Mission was founded on the traditional lands of the Gunggandji people, and subsequent state government policies resulted in the forcible relocation of First Nations and some South Sea Islander peoples to Yarrabah. The community is now self-governing under a Deed of Grant in Trust (DOGIT) land tenure status.

Yarrabah is now Australia's largest First Nations community. The 2016 census records the community as home to 2,559 First Nations' residents (33); however, Gurriny's regular client list of 3,600 in 2016 suggests that this is a significant undercount. Yarrabah was ranked amongst Australia's local government areas with the most extreme concentration of social and economic disadvantage (34). Associated with this disadvantage, Yarrabah experiences a high burden of chronic disease.

Until 2014, primary healthcare and emergency hospital services were provided in Yarrabah by the Queensland government-run Yarrabah Primary Health Care Centre. The centre was operated by one of Queensland Health (QH)'s Cairns and Hinterland Hospital and Health Service (CHHHS) with healthcare services offered by medical, nursing and health worker staff, and visiting community and allied health providers (35).

**Figure 2:**
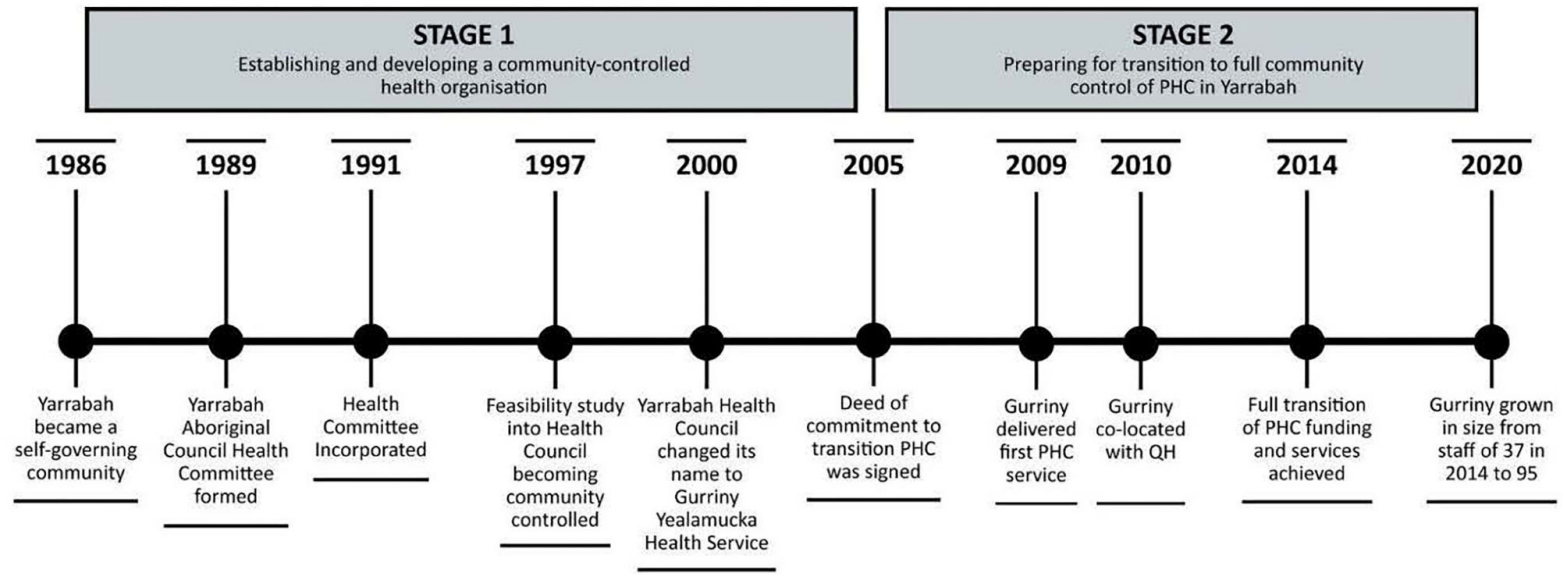
Stages of transition.

The first stage of the transition journey to establishing a community-controlled health organisation in Yarrabah was triggered by community dissatisfaction with the healthcare services provided by CHHHS in Yarrabah (see Figure 2). This led the Yarrabah Aboriginal Council to form a health committee in 1989, which was incorporated in 1991. A feasibility study in 1997 led to a renaming as the Yarrabah Health Council, and again in 2000 as Gurriny Yealamucka Health Service. The second stage (2005–2014) entailed preparing for transition to full community control of PHC in Yarrabah, with commitment to this end articulated through a Deed of Commitment between Gurriny and Commonwealth, Queensland and local government partners (2005) to achieve better health outcomes for Yarrabah. The four partners committed to implementing community control over the planning, prioritisation and management of PHC service delivery to the community of Yarrabah, and affirmed the essential requirements of community control as: (1) community identification of needs, aspirations and priorities; (2) a representative organisation based on good governance and best practise; and (3) a baseline document (Health Strategic Plan) for resource allocation (25). When the Deed of Commitment was signed in 2005, transition partners agreed upon the transition date of 2008, or 2010 at the latest.

In June 2014, the 28-year process was completed when Yarrabah became the first community in Queensland to transition PHC services from Queensland Health to First Nations' community control. Like other ACCHOs, Gurriny was funded through administratively complex funding arrangements through the Commonwealth government's Indigenous Australian's Health Program, primary health networks and Medical Benefits Schedule, QH program and grant funding, and other sundry funding such as research grants and philanthropic funds (36). The processes undertaken in these two stages are described more fully in Jongen et al. (25).

### Data Collection and Analysis

Interviews were held with people involved in the transition of PHC to community control in Yarrabah. A purposive sampling technique was initially used, with information-rich participants identified by senior managers at Gurriny and QH. These and self-identified participants were invited to participate in interviews that focused on their experiences of the transition, including enablers and barriers. A broad interview schedule (provided as a Supplementary Material) guided the interviews. As data collection progressed, theoretical sampling processes were used to identify further potential participants with diverse perspectives and ability to explore issues that had emerged from the initial data analysis.

Fourteen people were interviewed, eight of whom were First Nations' people. They included current Gurriny staff members (6, 3 of whom had previously worked for QH), ex-Gurriny staff members (2), current QH staff members (1), ex-QH staff members (3 in addition to those who were re-employed at Gurriny), other Yarrabah community members (1) and one other (1). With participant consent and at a place of their choice, face-to-face or telephone interviews were undertaken by SC and JM; interviews were audio-recorded and transcribed [for further details, see (25)]. Transcripts were given back for checking to those participants who requested them.

Eighty-eight historical organisational documents, dated from 2005, were provided by Gurriny as a point-in-time record of the transition, with augmentation of data from interviews of the retrospective viewpoints of those involved. The most common types of documents were progress or status reports (*n* = 19, 22%), published or internal reports (*n* = 11, 13%), plans (*n* = 10, 12%) and communication briefs (*n* = 9, 10%).

#### Data Analysis

The interview transcripts and Gurriny organisational documents were analysed using grounded theory methods. As described in Jongen et al. (25), the transcripts and documents were imported into NVIVO qualitative software and analysed using the constant comparison methods of grounded theory. Open-coding was conducted iteratively upon receipt of the transcripts and documents to identify actions and interactions (37). Codes that were associated in meaning were then grouped under higher order categories (38). These were integrated to determine the context, strategies implemented, and the barriers to and enablers of implementation (38). The strategies are described in Jongen et al. (25).

## Results

The enablers and barriers to transition are the factors that supported and/or hindered Gurriny's journey towards achieving community control of PHC during the two stages of transition. Three types of enablers and barriers were identified: internal factors within Gurriny's control, external factors directly related to the CHHHS handover which occurred largely outside of the control of Gurriny, and broader structural and policy factors outside the control of either Gurriny or CHHHS.

Internal barriers were Gurriny's lack of experience and capacity, and varying levels of community confidence. Internal Gurriny enablers were its leadership stability and capacity, community mandate, relationships with partner organisations, and ability to provide service continuity. External barriers were a lack of certainty, transparency and prioritisation of the transition process by CHHHS; systemic racism; difficulties obtaining and maintaining the necessary workforce; limited resources including insufficient, unstable or inappropriate funding support; and problems with information sharing. External enablers were CHHHS performance frameworks to keep transition progress on track. Broad structural barriers included an unsupportive Queensland government policy environment; government bureaucracy; and delays, conflicts and divisions. Broad structural enablers were high-level Commonwealth and QH government support and commitment, and funding (Table 1).

**Table 1:** Key barriers and enablers of the transition to community control in Yarrabah.

**Level**	**Barrier**	**Enabler**
Internal Gurriny factors	Gurriny's lack of experience and capacity	Gurriny's leadership stability and capacity
	Lack of community confidence	Community mandate
		Relationships with partner organisations
		Ability to provide service continuity
Relationships with CHHHS	A lack of certainty, transparency and prioritisation of the transition process by QH	Performance frameworks to keep transition progress on track
	Systemic racism	
	Difficulties obtaining and maintaining the necessary workforce	
	Limited resources including insufficient, unstable or inappropriate funding support	
	Problems with information sharing	
Broader structural and policy environments	An unsupportive Queensland government policy environment	High-level Commonwealth and QH government support and commitment
	Unresponsive government bureaucracy	Funding
	Delays, conflicts and divisions	

### Internal Gurriny Barriers

The two internal barriers were Gurriny's lack of experience and capacity and a lack of confidence by some community members in Gurriny's capacity to run an effective PHC service.

#### Gurriny's Lack of Experience and Capacity

By 2005, when the Deed of Commitment was signed by Gurriny, the Yarrabah Aboriginal Shire Council, QH and the Commonwealth Department of Health, Gurriny was still a small organisation, employing only 10 staff members. At the time, Gurriny Board members and some key senior managers had limited experience in health and some lacked financial expertise. There was a perception that the burden of transition was borne by Gurriny which had little funding, power or experience to enact the expected tasks. A Gurriny staff member noted:

“*the problem that we had all the way through, was that we were just a small organisation and we didn't have the capacity to just churn out all these things that Queensland Health were expecting us to churn out. And they were trying to measure us on our ability to provide that documentation or provide that evidence.”*

#### Lack of Community Confidence

Early in the transition process, some community members were concerned that the transition would incur a potential reduction in service availability and quality. Some local community members also expressed a lack of confidence in Gurriny's capacity to be in control of Yarrabah's health care. Some CHHHS staff were also community members, and they felt that they were already delivering quality services and achieving results, and that Yarrabah did not need community control. Community desire for community control was taken personally as it was related to direct criticisms of the work of CHHHS. A former-CHHHS/current Gurriny staff member reflected:

“*I could not see community control working. I was like, ‘… but we have everything. Why are we changing?”'*

### Internal Gurriny Enablers

The four internal enablers were Gurriny's: leadership stability and capacity, community mandate, relationships with partner organisations, and ability to provide service continuity.

#### Gurriny's Leadership Stability and Capacity

Gurriny's leadership was strong, stable and determined through the lengthy transition process. Gurriny had a reasonably stable Board throughout stage two of the transition years, which meant that experience and knowledge was retained. A Gurriny manager said:

“*I think that was really important having that consistent leadership at the Board level.”*

Senior managers and Board Directors played critical leadership roles in the oversight, guidance, planning and negotiating of transition processes with government, and built organisational capacity over a long timeframe. For example, all Board members partook in capacity building opportunities and an ex-officio Board member was recruited to bring financial expertise. Gurriny also had a dedicated Transition Manager, who was funded by the Commonwealth and responsible for transition coordination and program monitoring and reporting. Despite significant challenges, Gurriny leaders and staff demonstrated the leadership qualities of perseverance and determination to the extent that they were willing to do whatever was necessary to make the transition happen. Another Gurriny manager said:

“*All the way along, we did just keep chugging along, making the organisation better and smarter. We use[d] the deadline like with the 2010 Deed of Commitment. We all tried to use those deadlines to hold people to account, but we never thought that once we got to that deadline we'd just give up.”*

#### Community Mandate

The transition of PHC in Yarrabah to community control was driven by the dissatisfaction of community members about existing CHHHS healthcare service provision. A former Gurriny employee said:

“*there was quite a lot of people in Yarrabah… we had these great big ideas to develop community control because people weren't happy with the current services that was going on there*.”

#### Relationships With Partner Organisations

From the first stage of the transition journey, the implementation of evidence-informed programs and services was facilitated through research collaborations; researchers also evaluated their effects. The evaluations demonstrated to community and government stakeholders that Gurriny had built capacity, thereby enhancing confidence and trust, and helping to secure funding for workforce and leadership capacity development, employment of staff, and further expansion of programs and services.

Several of Gurriny's key alliances during the second stage of transition were within the ACCHO sector. The Queensland Aboriginal and Islander Health Council (QAIHC), the peak body for the Queensland state ACCHO, mentored Gurriny through high level strategic negotiations with state and Commonwealth governments and provided consultancy expertise. Gurriny was also able to share organisational knowledge, experience and resources with Apunipima Cape York Health Council (located in nearby Cairns) related to their simultaneous transition processes. They collaborated to develop community and research engagement strategies and shared in contracting various consultants to complete required planning and assessment tasks. Gurriny also established strategic partnerships through membership with the Northern Aboriginal and Torres Strait Islander Health Agency (NATSIHA) and National Aboriginal Community Controlled Health Organisation (NACCHO) to leverage knowledge and support to progress the transition. A Gurriny manager said:

“*we leaned a lot on expertise that might've came our way from QAIHC and from Apunipima – just to tap into other work that they'd already done, or talking to different people. And that was kinda how we got through it.”*

Finally, partnerships and alliances with consultancy services provided strategic, legal and operational guidance to progress the transition. For example, an Organisational Capacity Review, developed by consultancy firm Bentley's, identified core strategy areas requiring improvement as: workforce planning and development, the service delivery model, information technology, finance/funding modelling, and legal issues and governance (32). A Gurriny manager recalled:

“*Bentley's came in and done a twelve-month review on Gurriny. From the Board right down to service delivery, to IT and workforce, all of that. And out of that were some really good stuff because there were some things that we did lack. And I thought, ‘wow, I didn't realise that.”'*

The Bentley's Organisational Capacity Review helped to create clarity and certainty concerning government expectations and requirements, was incorporated within Gurriny's plans, strategies and actions to progress transition, and enabled Gurriny to take appropriate action.

#### Ability to Provide Service Continuity

Planning processes to develop a health services plan and delivery model were focused most intensively in the years 2006–2008. In 2007, external consultants provided a Proposed Service Delivery Framework for Gurriny, including recommendations about services that should be included, and the integration of clinical services with previously established social and emotional well-being programs. Gurriny and CHHHS also developed a Yarrabah Health Services Plan (2008) based on the assessment and mapping of Yarrabah's health service needs and options; this became a guiding document for service provision. Later in the transition process, health program planning occurred annually. Despite workforce supply challenges, Gurriny achieved its staffing requirements and was able to provide continuous services during transition.

### External Barriers in the Relationship With CHHHS

The five barriers that were beyond the control of Gurriny but were apparent in its relationship with CHHHS were a lack of certainty, transparency and prioritisation in the transition process; systemic racism; difficulties obtaining and maintaining the necessary workforce; limited resources including insufficient, unstable or inappropriate funding support; and problems with information sharing.

#### Lack of Certainty, Transparency, and Prioritisation of the Transition Process

Being the first Queensland transition of PHC from government to community control, there was a general lack of clarity and understanding amongst all involved parties about the process, and a lack of expert knowledge and guidance or frameworks to guide the transition. Leaders within CHHHS did not have the relevant expertise, experience or resources to oversee the process, and many stakeholders were not aware of which legislation and government policies and procedure could affect transition. Frequent changes in these laws, policies and procedures meant that policy was developed as the transition occurred. A former CHHHS manager said:

“*we had this unprecedented industrial arrangement where we then had to question how staff would transition from one service to another… it was the first time it had happened, so policy was kind of being developed as it was happening.”*

A Gurriny manager also reflected:

“*I don't know even if the politicians even understood that they might have some legislation or some policies that are actually gonna stop or impact on what they're saying they want done.”*

Furthermore, the transition process comprised one small component amongst other competing priorities of the CHHHS portfolio, which resulted in its low prioritisation. There was no dedicated leadership within CHHHS to oversee and progress the transition. A former CHHHS manager recalled:

“*…the first failing. This is a multi-million-dollar procurement over a significant period of time. And in any other procurement of this size, you would actually have allocated a person managing that. So it was one of those things that got managed when it came up. When there was a need for it to come up, it came up and the rest of the time, to be honest, it wasn't something that we had somebody who made it their full-time priority.”*

#### Systemic Racism

Systemic racism manifested from the start of the transition process in the form of resistance, negative reactions and a lack of support from some CHHHS staff, and through the inherent power imbalance of the two organisations and risk-averse processes of CHHHS. A former Gurriny manager said:

“*originally when we finished the Feasibility Study report [1998] and we gave a copy to the state government, and the state government services was upset. So all the [QH] nursing staff was really upset and they sort of rebelled. The government itself didn't accept the Feasibility Study report.”*

This resistance and reluctance to relinquish control continued throughout the transition. Presenting as a catch-22 situation, it was based in the (not unfounded) concern about Gurriny's relative lack of experience and capacity to manage the complex operations of the large PHC service to provide quality care to ameliorate the considerable burden of disease in the community. A former CHHHS manager also noted:

“*you had many people playing the politics of ‘this is community driven and led.' Like I agree in the principle, but if you're going to give it to people that actually understand health and have some skills and knowledge I think. ‘Cause there's risks behind that if you don't.”'*

From 2009, this reluctance to let go of control contributed to a shift in commitment from community control to an explicit focus on service integration through co-location of Gurriny and CHHHS. Gurriny struggled to secure CHHHS commitment to a plan and timeline for full transition. A Gurriny manager noted:

“*I did feel a little bit that Queensland Health… weren't that willing to let go.”*

The marked power imbalance that existed between QH/CHHHS and Gurriny was also seen as a barrier to an equitable partnership between the two organisations and a significant source of systemic racism. A former CHHHS manager perceived:

“*We held the power in this relationship. There's no questioning that.”*

The inequality in power was evident, for example, in the risk-averse service Operating Deed (2016) which set out the legal relationship between the two services. The Deed and Lease agreements, which were written to protect QH's reputation and funding, demonstrated a lack of trust by CHHHS in Gurriny's capacity to take control of PHC services. The Operating Deed was described by a Gurriny staff member as “*risk-averse, protective, hand-holding, unilateral, paternalistic and overbearing*.” For example, despite only 20% of the services being funded by QH (the balance being funded through Commonwealth grants and Medicare), the Operating Deed required Gurriny to account to CHHHS with data and reports for every aspect of service delivery. A former manager from Gurriny shared this perspective:

“*The Deed of Operations… was incredibly one-sided, judgmental and demanding from the Queensland Health side. And absolutely, when you considered they were providing less than twenty per cent of our funds, they were wanting all the data set, all of the knowledge… when in fact, when you look at the amount of money that was coming into Yarrabah at that time for the Health Services, Gurriny got a tiny drop in the ocean of that.”*

Through these documents and other indications, participants inferred an implicit message on the part of CHHHS that they expected Gurriny to fail, and that CHHHS would need to reassert control. In parallel, the willingness of QH/CHHHS staff to support Gurriny in building the required knowledge and capacity varied. A Gurriny manager said:

“*the hidden message underneath that was, ‘we're gonna keep tentacles involved in this because they're probably gonna fall over and we'll have to step back in.”'*

As a result, Gurriny levels of reciprocal trust in the goodwill of CHHHS fell. For example, a Joint Working Group was reported as difficult to progress due to the “*risk… that QH will railroad (Gurriny's) work according to their needs*.” Gurriny documents also reported that a joint planning, monitoring and reporting framework was put on hold because required support from CHHHS was not provided; the Transition Risk Management Plan couldn't progress due to lack of cooperation from CHHHS; a Transition Implementation Plan that was supposed to be jointly developed faltered due to lack of CHHHS involvement; and a review and design framework to support joint accreditation was difficult to progress in collaboration with CHHHS.

#### Difficulties Obtaining and Maintaining the Necessary Workforce

The transition of CHHHS staff across to Gurriny entailed an unprecedented industrial dilemma and major challenge in the transition process. As public servants, CHHHS staff experienced better employment conditions than most of the private sector workforce, and some CHHHS staff were concerned about the potential that they might lose their jobs, accrued benefits and leave entitlements. Differences in organisational cultures and values, models of care, and staff award wage and entitlement systems between government and non-government systems meant that not all positions in CHHHS were to transition to equivalent roles. Also, a strong resistance from CHHHS staff contributed to their unwillingness to work for Gurriny.

At the 11th h and without consultation with Gurriny, CHHHS/QH decided to offer redundancies to their Yarrabah staff members. However, the conditions of the redundancy offer meant that those who accepted would need to wait for 3 months before they could apply for available positions at Gurriny. Gurriny responded by temporarily employing people for that 3 month period to enable CHHHS staff to apply, which impacted on their capacity for service continuity and achievement of a smooth transition. A Gurriny manager recalled:

“*it was a real pain but we worked out if they were made redundant… they had to not work for three months… so what we could do is, we would only put on staff for three months to fill positions, to keep the wheels chugging along and then we would advertise the permanent positions and if a Queensland Health staff was interested in applying, they were welcome to apply. There was no guarantee they'd get the job but we would hold off on recruiting permanent positions until they were eligible to apply. Which is what we ended up doing.”*

Although Gurriny managers supported the transition of CHHHS staff through engagement and providing the opportunity to apply for positions, ultimately, only two former CHHHS staff out of Gurriny's staff complement at the time of 37 transitioned across to Gurriny.

#### Limited Resources Including Insufficient, Unstable or Inappropriate Funding Support

There were three funding phases relevant to the transition: (1) the pre-transition operating costs, which were borne by CHHHS; (2) the costs of the transition process itself; and (3) the ongoing operating costs post-transition which were borne by Gurriny. The method for calculating the funding to be transferred was a key barrier to the smooth transition of PHC responsibilities in Yarrabah.

There was a lack of clarity by CHHHS pre-transition about the what the costs of delivering PHC services to Yarrabah entailed. Related to this, the type of services and amount of funding that would be transitioned from CHHHS to Gurriny were unclear throughout much stage two of the transition process. There was no assessment to inform the funding decisions of community needs and demand for services, the actual services Gurriny would deliver, the cost of Gurriny's model of care, or potential service delivery improvements.

Transition costs included the costs of infrastructure, accreditation, recruitment, systems, developing pathways and models of care. Limitations in the availability of resources to progress organisational development, and uncertain or unstable funding hindered Gurriny's workforce and organisational growth throughout the process. For example, Gurriny was required by Commonwealth and state governments to complete various planning processes that necessitated the engagement of external consultants, and frequently no additional funding was allocated for these efforts.

The costing method used by CHHHS to determine the funding they would transfer upon final transition was driven by their (non-stated) vested interest in managing a cost neutral transition of PHC services (i.e., they would continue to contribute the same funds as they had previously expended - regardless of actual cost of service delivery or growth). The budget was based on actual expenditure which was lower than the operational budget (presumably due to unfilled positions, and the delivery of less service provision than that planned/budgeted for). Furthermore, payment was to be provided after service provision. Funding was coming directly from the CHHHS budget and they had competing needs and priorities. This funding approach was not anticipated by Gurriny and was considered by participants from both services to be inappropriate. A Gurriny manager said:

“*If someone could come back and say, ‘well actually… you are delivering more care than was anticipated when we gave you this small amount of money. This is actually what it costs, and this is what you should be funded for to deliver that care…' because Queensland Health said, ‘well these are the positions: four nurses and a number of Health Workers. You will deliver this, this and this.' But we're doing triple that amount of work on whatever that budget is.”*

A former CHHHS manager explained:

“*There was a very strong drive from the (hospital and health service) … that we weren't going to give Gurriny any more money than we actually would save by not providing that service… and we commissioned that audit…. So I suspect that a conservative approach may have been taken.”*

Furthermore, the final funding amount was decided almost immediately prior to the official handover. This meant that Gurriny was compelled to plan service delivery without any clarity about the available level of funding for those services. A former CHHHS manager said:

“*Gurriny didn't know how much money they were getting, they didn't know what services they could offer, so they couldn't have positions in place, ready to fill, to go into a transparent recruitment process.”*

After the official handover of funding and services in June 2014, funding issues continued to plague Gurriny's capacity to provide healthcare. For the first 3 months, CHHHS did not pay the allocated funding to Gurriny or respond to the invoices sent. This meant Gurriny did not have the required funding for 11 positions. Furthermore, once funding commenced, CHHHS paid at the end rather than the beginning of the month. These issues created a significant financial burden and compromised the solvency of Gurriny in the first year following transition. A Gurriny manager said:

“*Queensland Health … didn't pay their first monthly remittance for those positions until the September of that year. So Gurriny was almost pushed to bankruptcy because they had once again, [acted in] good faith and employed people, but the funds weren't there because Queensland Health didn't pay.”*

#### Problems With Information Sharing

Issues related to the sharing of client information between Gurriny and CHHHS were a significant and persistent barrier to successful service collaboration over many years. For example, the CHHHS CEO agreed to share medical records while the services were co-located. A Gurriny staff member recalled:

“*it was constant head-butting right up til twenty thirteen when… (the) CEO of Cairns Hospital at the time said, ‘enough is enough. We cannot go on with these separate records. We must have one record because we continue to compromise patient care.”'*

However, following this decision, the Nurses Union advised CHHHS nurses to only use paper records. To this day, Gurriny's client information system is shared with CHHHS emergency department doctors in Yarrabah, but CHHHS emergency department nurses do not share their client data.

### Enablers in the Relationship With CHHHS

The two external enablers in the relationship with CHHHS were funding and performance frameworks to keep transition progress on track.

#### Performance Frameworks to Keep Transition Progress on Track

A package of performance frameworks was prepared by Queensland Health and used throughout the transition process to keep Gurriny and CHHHS on track in key action areas of the transition. These included a Strategic Policy Framework for Transition, a Readiness Assessment Framework, Industrial Relations Guidelines, Information Management Guidelines, Joint Communication and Engagement Guidelines, Evaluation Guidelines, and Funding Guidelines. The performance frameworks helped Gurriny to assess its implementation of core strategies across all areas of its operation and to demonstrate organisational capacity to operate a complex PHC service. This was a requirement of government stakeholders and necessary for building trust and securing ongoing support for transition.

### Broad Structural Barriers

The three broad structural barriers were an unsupportive policy environment; government bureaucracy; and delays, conflicts and divisions.

#### Unsupportive Policy Environment

The lack of dedicated CHHHS leadership and resources (discussed above) largely resulted from the difficult funding and policy environment that shaped the QH organisational context, capacity and priorities at the time of transition. The transition process occurred during a conservative state government term. Funding cuts compelled QH to reduce staff contingents and CHHHS was under considerable pressure to allocate all resources to frontline service delivery. It was very unlikely in this environment that it would have been achievable to acquire a dedicated position to manage the transition. A former CHHHS manager noted:

“*I don't… in retrospect think that we would actually have been given approval to have somebody dedicated to work on this. It was a very difficult time to get administrative staff employed because of the philosophy of the Newman government and the caps that it had set on employment. And the head-count reduction… it was trying to achieve.”*

#### Unresponsive Government Bureaucracy

A lack of capacity for reflexive, innovative and creative responses in government bureaucracy stalled and complicated decision making, hampering effective collaboration between Gurriny and CHHHS. Decisions went to and fro between sub-committees and lawyers to the extent that those involved could no longer make sense of the process. This unresponsive bureaucratic system not only disempowered Gurriny, but also diminished the decision-making ability of managers and leaders in CHHHS to seek and implement creative solutions. A Gurriny manager reflected:

“*I think sometimes when people get into middle management or upper level management in bureaucracies… they just can't make decisions, so they deflect that decision across to a sub-committee that'll look at it for six to twelve months and it drifts into the ether and gets lost in translation.”*

There was a disconnect between implementation on the ground and the support and policy directives coming from top levels of government. Another Gurriny manager reflected:

“*One of the barriers was that it seemed at like the really high levels of government… they seemed to support this idea but when you got down to the bureaucrats who were supposed to do it… they then didn't know how to do it.”*

#### Delays, Conflicts, and Divisions

Delays in the transition process were noteworthy and a significant hindrance. Prior to setting an official handover date in 2014, delays and setbacks in the transition process were so frequent that many participants considered that it was not getting anywhere. The transition was conditional on Gurriny's completion of ever-shifting deliverables, and was made even more difficult by limited resources and cooperation. The sheer quantity of work the transition required [see (25)] also contributed to the delays. A former CHHHS manager said:

“*The transition for Yarrabah just had been coming for a very long time and it just dragged on and it dragged on and it dragged on, and it got to a point that many staff believed it would never happen.”*

Conflicts and divisions experienced between Gurriny and CHHHS were particularly evident during the years of co-location (2010-14) when the relationships between Gurriny and CHHHS staff were fractured. There was a distinct separation between the two organisations, with no apparent collaboration, despite being co-located in the same building. A Gurriny manager reflected:

“*I think what had happened was that when we co-located back in 2010… they tried to merge teams and they had two sets of Line Managers and you know, it was just really unpleasant. It was actually creating some little fires and there was lots of assumptions and toxic kind of team dynamics and things going on here all the time.”*

### Broad Structural Enablers

The two key structural enablers identified were funding and the support and commitment to transition at high levels of government.

#### Funding

State and Commonwealth government funding commitments were essential to transition. By 2005, when the Deed of Commitment was signed, the receipt of sustained Commonwealth government funding for the first Transition Officer position and four permanent social and emotional well-being positions was a defining moment for Gurriny, helping to create stability and sustainability in the organisation. A community research partner said:

“*by 2005, the Commonwealth Office of Aboriginal Health actually came to the party and offered the first four permanent positions for Gurriny.”*

#### High-Level Government Support and Commitment

Many of the enablers of the transition were at least partly a result of the broader state and Commonwealth government structural and policy systems. The support and good will of high-level QH and the Commonwealth Department of Health and Ageing bureaucrats towards the transition, evidenced by their signing of the Deed of Commitment, was identified as an enabler. Many high-level CEO to CEO meetings, committee meetings, Transition Steering Committee meetings, and clinical leadership meetings were held between Gurriny and CHHHS to support the transition and address operational issues. These pertained to issues regarding information systems, the signing of a memorandum of understanding between doctors, the use of a consent form, and the sharing of medical record systems. Despite protracted delays in dealing with many issues, key individual leaders within QH/CHHHS demonstrated courage in resolving them through taking charge of situations that had reached an impasse.

In 2014, an 11th-h Ministerial directive for setting and publicly declaring a clear transition date clinched the commitment, investment of resources, and accountability from government stakeholders, and resulted in rapid progress in the transition process. A Gurriny manager said:

“*It was … the public declaration of a date that actually spurred everybody into action.”*

Once transition had been achieved, Gurriny leaders implemented systems to enable delivery of a more comprehensive primary healthcare service to the people of Yarrabah.

## Discussion

**Figure 3:**
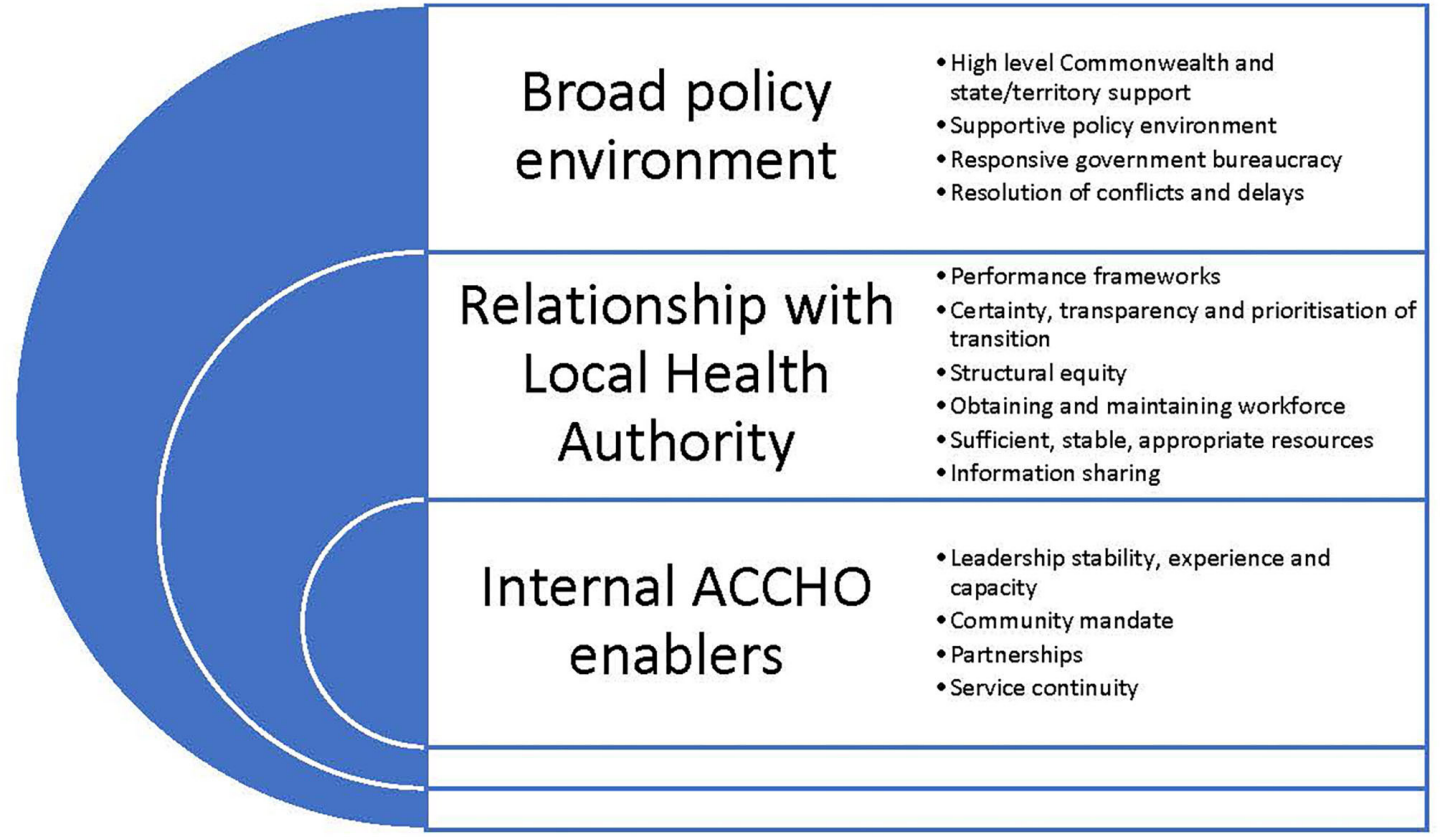
The enablers of transition.

This paper sought to identify the barriers that hindered and enablers that supported the transition of PHC services to First Nations' community control through Gurriny in Yarrabah. The experiences of Gurriny demonstrate that, as in other PHC transitions in other parts of Queensland and the Northern Territory, there are a range of factors which can both hinder and support the process (28, 39). In Yarrabah, these occurred at three levels: factors within Gurriny itself, those that were directly related to the handover from CHHHS, and broader structural and policy factors outside the control of Gurriny or CHHHS. The enablers of transition, extrapolated from the case of Yarrabah's transition, are depicted in Figure 3.

Internal Gurriny organisational barriers and enablers played a role in the transition. The mandate from the community to pursue self-determination regarding their own healthcare and other services drove the transition to community control, although work was required by Gurriny to strengthen community confidence. Strong organisational leadership and good governance were critical enablers of community control, with ongoing capacity development undertaken at the community, organisational and clinical levels (40). The dedication and perseverance of Gurriny leaders in efforts to achieve the vision of community control also contributed to the achievement of the 2014 transition. Partnerships and collaborations with research, ACCHO and other organisations also strengthened the capacity of Gurriny (39).

Most of the barriers to transition were directly related to the state government handover of control from CHHHS to Gurriny. As in examples of other transition processes, whilst the act of transitioning to community control was based on Commonwealth and state governments' commitment to decolonising health service provision, key among the challenges was the imbalanced power relationship and the reluctance of CHHHS to relinquish control (8). This reluctance to cede control to First Nations organisations is founded on a history of conflict and resentment between government health departments and ACCHOs (41)—and comprises an example of systemic racism [(28), p. 58]. In Yarrabah, as for other transitions, there were perceptions that CHHHS did not acknowledge the cultural legitimacy of Gurriny, imposing unreasonably high levels of accountability, micro management and reporting [(28), p. 58].

The transition encompassed a challenging cross-cultural process between two very different organisations with different values and priorities (28). CHHHS had concerns about Gurriny's capacity and, as in other transitions of PHC to community control, Gurriny did considerable work to address government doubts (28). Some government stakeholders perceived the transition to the ACCHO as an implicit criticism of existing services, especially relating to access and appropriateness (28). Factors such as a lack of trust arising from the different interests of partners across government and community sectors, the cross-cultural nature of relationships and a perception of underlying racism influenced different organisational perceptions of priorities, meaning, values, interests, goals, choices, expectations and timelines. There was a “mutual perception of failure to maintain commitment and a sense of significant pressure on established relationships and mutual trust” (28).

In part, the CHHHS resistance to community control came from the concerns of Yarrabah-based QH staff about the practicalities of transitioning their employment arrangements to Gurriny. For many QH staff, there was a sense of ownership over the clinic and lack of comfort about working for Gurriny. As in other transitions, government staff concerns included fears of losing job security, wages, and salary-related benefits, and possible fears about marginalisation and changes in accountability relationships (28). Industrial relations issues around transferring existing employees were complex. The perceptions of staff in other PHC services experiencing transition were bluntly articulated thus: “the opposition of local non-Indigenous government staff was deeply rooted in disbelief in the capability of Aboriginal people and a distrust of Aboriginal organisations: ‘I'll say blunt on record… that they don't want to work for a black organisation”' (28).

Inadequate resources, inflexible funding arrangements and the absence of clear agreements about funding amounts, conditions or timeframes were key barriers to a smooth transition of PHC responsibilities in Yarrabah (28, 40). Due to the poorer health status of the Yarrabah population, it was reasonable to expect greater (at least double the average per capita) government expenditure than for non-First Nations Australians in order to achieve equity of access (17). However, the 11th-h funding package was based neither on current or projected health need, demand for services, or equity (13). Instead, the funding allocation at transition was based on historical expenditure and designed to be cost neutral for CHHHS. Furthermore, while funds pooling was an explicit intention of transition, there was no substantial work from government towards achieving those aims. It became apparent that if transition was to make a significant difference to health outcomes, additional investment from the Commonwealth and Queensland governments would be required.

A package of performance management framework documents was developed by Queensland Health to assess the competencies and capabilities of Gurriny's governing board and its organisational readiness, and was applied as a condition of approval of transfer. Similar to other PHC transitions, standards were high, with Gurriny staff noting that CHHHS services were not similarly scrutinised (28). As in other transitions, it was “perceived by some in the ACCHO sector to be an extension of a generally excessive risk intolerance displayed by both levels of government” and represented a deficit approach to community control—a complicated process intended to mask the real underlying thought that “blackfellas can't run these things” (28). This type of risk intolerance in funding Aboriginal organisations meets the definition of systemic racism, as it has a differential application and impact in First Nations communities and organisations (8).

Despite continuing government policy commitments to community control, there was nevertheless “no enduring basis for accountability by governments for … transferring PHC provision to community-controlled healthcare providers” (26). Whilst Commonwealth funding support was a critical early enabler for the transition, there was insufficient recognition of the need for adequate resourcing of the change process itself [(28), p. 6]. Broad changes of national and state elected governments, restructuring of QH and reallocation of health service delivery responsibility to regional Hospital and Health Services in 2011-2012, led to frequent shifts in government priorities, policies and personnel, and high-level government prioritisation of transition was withdrawn as the transition progressed. The focus was shifted from community control to “service integration” (40). As for PHC transition processes in other communities, the broader structural enablers in Yarrabah included a strong early commitment, policy support, and goodwill from governments towards the vision of community control (8, 28).

There has been policy commitment to community control in Australia for more than 30 years (8). But transitions of PHC to community control in Yarrabah and other communities have been “difficult and complex, and progress has been slow” (28). Despite consistent policy support, there was a failure at the outset to negotiate and secure the required level of authorisation and commitment to transitioning PHC services to community control (7), and an apparent reluctance of government health administrators to engage effectively with and transfer control to Aboriginal communities (8). Implementing community control takes time, and sufficient support and resources are required to navigate the process successfully (25, 26). Efforts to transfer services to community control have generally been conducted under tight timelines, which are inappropriate for the needed long-term commitment (26). The slowness of progress in the case of Yarrabah created a perception that there was limited government commitment to expected timelines and processes. As in other PHC transitions, these challenges manifested in delays, divisions and conflicts and a lack of certainty and clarity in the process and created opportunities for divisiveness and blame allocation.

Queensland Health now has a reform agenda to embed health equity across the health system and address the legacy of systemic racism described in this case of PHC transition. For the first time, a legislative requirement was passed by the Queensland Parliament in August 2020 which embeds a requirement for each Hospital and Health Service (HHS) to redesign and reorient local health systems to better listen to and support First Nations Queenslanders, address historical and ongoing economic and social injustices, and recognise First Nations sovereignty and right to self-determination (42). As part of this agenda, a relocation of authority and control from governments back to Indigenous organisations is needed to provide governance of First Nations peoples' right to self-determination that originates from their inalienable connexions to lands, waters and the natural world (31). The preference of First Nations people to access ACCHOs over mainstream PHC services supports this agenda (43, 44). For example, one study of patient access to one urban and five regional Queensland ACCHOs found that First Nations people preferred using the ACCHOs over mainstream PHC services (43). Furthermore, ACCHOs in Queensland have achieved very high access rates, with 60–100% of First Nations people who live close to ACCHOs accessing their services (44). Such control over essential services is recognised as an underlying social determinant of health and a health intervention in its own right (45, 46).

## Limitations

This research is based on the perspectives of 14 participants who retrospectively recalled the enablers and barriers 4 years after the actual transition occurred, and the analysis of 88 point-in time documents. Although participants were selected based on their roles in the transition and/or unique perspectives, efforts to interview QH staff were met with limited success. Of the 12 current or former QH staff invited to interview, only 3 accepted. In contrast, of the 12 current or former Gurriny staff members invited, 8 accepted. Similarly, our analysis of historical organisational documents was based on documents provided by Gurriny. Similar documents from Queensland Health could not be assessed because acquiring access to Queensland Health documents required additional ethical approval which was not possible within the time limits of the research.

## Conclusions

The implementation of community control in Australia requires commitment at three levels: by the local community organisation, in the relationship with the government health authority, and at the broader Commonwealth and state structural and policy level. The transition of PHC to community control in Yarrabah took 28 years. It was complicated by the ACCHO's lack of experience and capacity, wavering community confidence; the local government authority's lack of certainty, transparency and prioritisation of the transition process; systemic racism; difficulties obtaining and maintaining the necessary workforce; limited resources including insufficient, unstable or inappropriate funding support; problems with information sharing; and the broad structural and policy barriers of an unsupportive policy environment; government bureaucracy, delays, conflicts and divisions. Enablers were community-controlled leadership stability and capacity, community mandate, relationships with partner organisations, ability to provide service continuity, CHHHS performance frameworks to keep the transition process on track, and Commonwealth and Queensland government funding and high level support and commitment. This case example of a transition from government to community control of PHC highlighted the ongoing power issues that are faced every day by community-controlled organisations that co-exist with mainstream health systems within a colonial power structure.

## Data Availability Statement

The original contributions presented in the study are included in the article/Supplementary Material, further inquiries can be directed to the corresponding author.

## Ethics Statement

The studies involving human participants were reviewed and approved by Ethics Approval was received from Far North Queensland Human Research Ethics Committee (HREC/18/QCH/95 HREC/18 Project ID: 41295). The patients/participants provided their written informed consent to participate in this study.

## Author Contributions

JM contributed significantly to the conception and design of the research project and wrote the paper. CJ was responsible for completing the grounded theory analysis of organisational documents and participant interviews, and for writing the first draught of the results. SC was the project manager and completed the ethics applications. CJ, SC, and JM all contributed to the development of interview questions and SC and JM conducted interviews with participants. RF, KP, and SA were all members of the reference committee, helped to source documents and recruit participants, provided guidance on the evaluation, and provided feedback on the final manuscript. All authors read and approved the final manuscript.

## Funding

The evaluation of transition to community control in Yarrabah was funded by Queensland Health. QH was represented on the evaluation advisory committee, but played no role in the design of the study or collection, analysis and interpretation of data or in the writing of the manuscript.

## Acknowledgements

The authors acknowledge all participants for their willingness and time to be interviewed and the Reference Committee for guiding this project. Reference Committee members included: Venessa Curnow (Director, Aboriginal and Torres Strait Islander Health Unit, CHHHS, Queensland Health), Vanda Simpson (A/Manager, Aboriginal and Torres Strait Islander Health Branch, Strategy, Policy and Planning Division, QH), Gregory Richards (Aboriginal and Torres Strait Islander Health Branch, QH), Sue Andrews (CEO, Gurriny), Ruth Fagan (Business Manager, Gurriny), Karen Dennien (Director of Operations, Gurriny), Kingsley Pearson (Senior Medical Officer, Gurriny), Chris Doran (Health Economist, CQU), Sandra Campbell (Research Project Manager, CQU), Janya McCalman (Senior Research Fellow, CQU). We also acknowledge Professor Roxanne Bainbridge from the Gungarri/Kunja nations and Professorial Research Fellow at Central Queensland University for her involvement and guidance in the early development of the evaluation. We also wish to acknowledge Ros Calder for editing the manuscript.

## Conflict of Interest

RF, KP, and SA are managers at Gurriny. The remaining authors declare that the research was conducted in the absence of any commercial or financial relationships that could be construed as a potential conflict of interest.

## Publisher's Note

All claims expressed in this article are solely those of the authors and do not necessarily represent those of their affiliated organizations, or those of the publisher, the editors and the reviewers. Any product that may be evaluated in this article, or claim that may be made by its manufacturer, is not guaranteed or endorsed by the publisher.

## Abbreviations

PHC: Primary healthcare
ACCHO: Aboriginal Community Controlled Healthcare Service
Gurriny: Gurriny Yealamucka Health Service
QH: Queensland Health
CIHER: Centre for Indigenous Health Equity Research
CQU: Central Queensland University
CHHHS: Cairns and Hinterland Hospital and Health Service
QAIHC: Queensland Aboriginal and Islander Health Council
DOGIT: Deed of Grant in Trust
SEIFA: Socio-Economic Indexes for Areas
NATSIHA: Northern Aboriginal and Torres Strait Islander Health Agency
NACCHO: National Aboriginal Community Controlled Health Organisation.
