# Supplementary material for: The Barriers and Enablers of Primary Healthcare Service Transition From Government to Community Control in Yarrabah: A Grounded Theory Study
Source: Front Public Health. 2021 Oct 14;9:616742. doi: 10.3389/fpubh.2021.616742 (PMC8551548; doi:10.3389/fpubh.2021.616742)
Supplement: Supplementary file 1 [file Data_Sheet_1.PDF]

Document overview

A total of 88 Gurriny documents created or published between 2005-2017 were reviewed (Figure 1).

Figure 1: Document Types

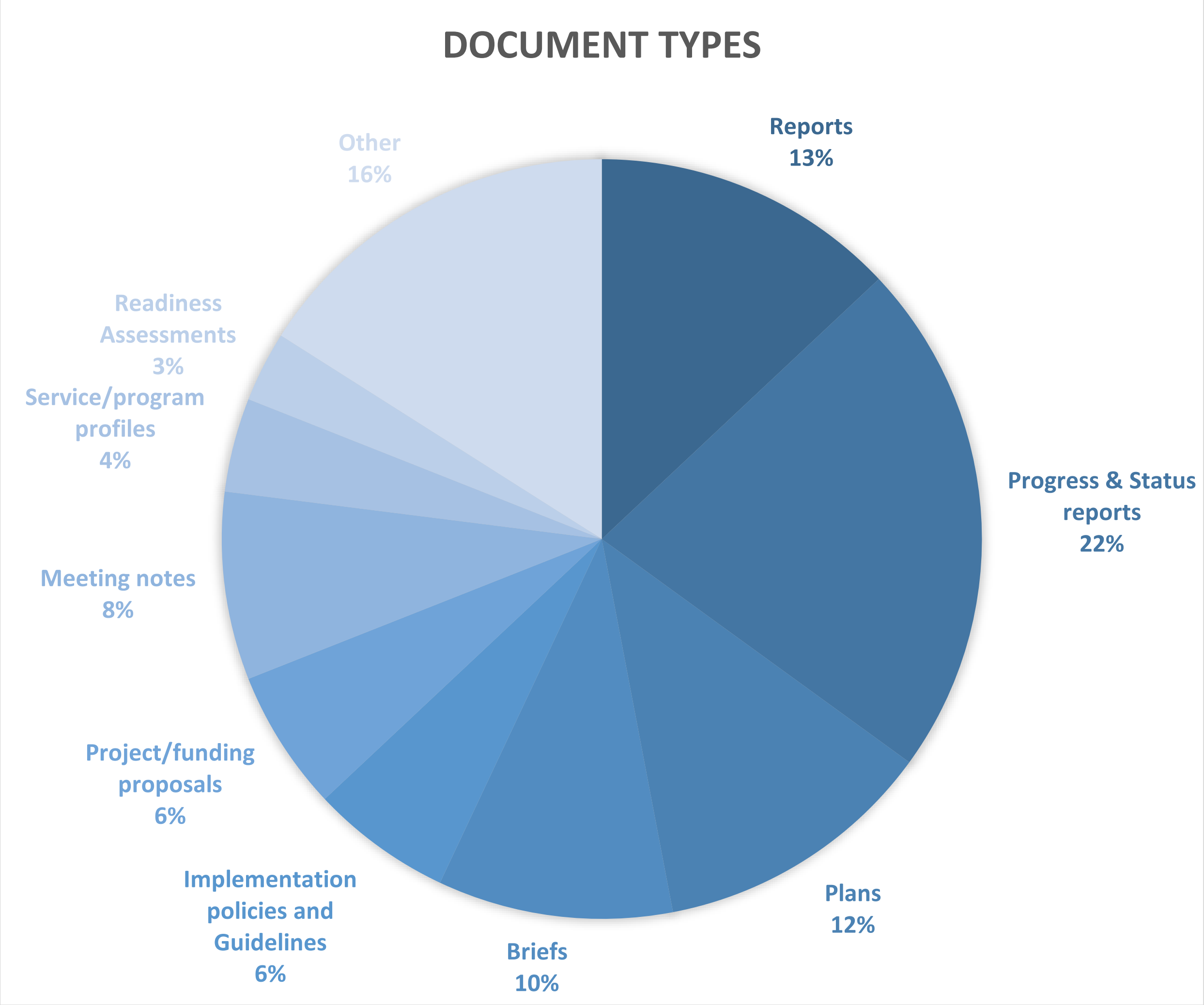

Document Type	Document Name	# Documents
Report	• The Practice Health Atlas (PHA) Gurriny Yealamucka Health Service (2013)<br>• The Practice Health Atlas (PHA) Gurriny Yealamucka Health Service (2012)<br>• Eagar K and Gordon R (2008). Access and equity - the funding required to close the gap in Aboriginal and Islander health in Far North Queensland. Centre for Health Service Development, University of Wollongong<br>• Bentleys (2014). Organisational Capacity Review Report (draft)	Total = 11<br><br><u>By year</u><br><br>2007 = I<br>2008 = III<br>2010 = I<br>2012 = I<br>2013 = I<br>2014 = II<br>2015 = II

	• Bentleys (2015). Organisational Capacity Review Report (final)<br>• Yarrabah Health Reform Project: Progress report on the project implementation from September 2006-July 2008. Ross Andrews, Transition Manager (2008)<br>• Belbin (2007). Gurriny Yealamucka Transition Project Talent Management<br>• McCalman, J., & Jones, G. (2015) Gurriny Yealamucka Health Service Evaluation Report 2014-15. Cairns: The Cairns Institute, James Cook University.<br>• Report – Pathways to Community Control, 2008<br>• QH report on assessment of funding to be transitioned, 2014<br>• Gurriny internal report, discussion paper on model of care, 2010<br>•	
Plan	• Risk management action plan (Bentleys), 2014<br>• Specifications for the Transition Implementation Plan, 2006<br>• Readiness Tool Action Plan, 2014<br>• Final transition action plan (2013 – Isn't this a progress report though?)<br>• Transition plan draft, 2012<br>• Strategic Plan 2011-2013<br>• Annual performance and accountability Strategic Operational Plan 2010-2011<br>• Transition Plan, 2008<br>• Plan for PHC model and transition implementation, 2010<br>• QH/Gurriny Communication and Consultation strategy, 2010 ???	Total = 10<br><br><u>By year</u><br>2006 = I<br>2008 = I<br>2010 = III<br>2011 = I<br>2012 = I<br>2013 = I<br>2014 = II
Brief	• Transition brief, 2015<br>• High level transition brief, 2014<br>• Reporting brief, readiness tool, 2012 (x2)<br>• Transition brief to board of directors, 2009<br>• Brief – Status Report for Partnership, 2009 (x2)<br>• Brief – Status Report for Partnership, 2010<br>• Brief from CHHHS and Gurriny CEO's to QH Director General, 2009	Total = 9<br><u>By year</u><br>2009 = IIII<br>2010 = I<br>2012 – II<br>2014 = I<br>2015 = I
Progress Report	• Transition unit report, May 2013<br>• Transition unit report, June 2013<br>• Transition unit report, Sept 2013<br>• Transition unit report, Dec 2013 & Jan 2014<br>• Transition unit report, Feb & March 2014<br>• Transition unit report, Feb & April 2014<br>• Transition unit report, May 2014	Total = 8<br><br><u>By year</u><br>2013 = IIII<br>2014 = IIII

	• Gap Analysis, Progress Report, 2013 (x2 or just repeat?)	
Performance Framework	- Performance framework table<br>- Jan – Jun 2013<br>- Jan – Mar 2014<br>- April – June 2014<br>- Jul – Sep 2014<br>- Oct – Dec 2014<br>- Jan – Mar 2015<br>- Apr – Jun 2015<br>• Reporting deliverables for transition project, June to July 2015	Total = 8<br><br>By year<br><br>2013 = I<br>2014 = IIII<br>2015 = III
Meeting Minutes	• High Level T2CC Committee Meeting (x3), 2014, 2014, 2015<br>• Transition Plan Briefing Notes (progress report) (2006)<br>• Transition Committee meeting minutes, 2008<br>• Transition Committee meeting minutes, 2009 (x2)	Total = 7<br>By year<br>2006 = I<br>2008 = I<br>2009 = II<br>2014 = II<br>2015 = I
Implementation policies and Guidelines	• Evaluation Guidelines, 2013<br>• Readiness Assessment Framework, 2013<br>• Information Management Guidelines, 2013<br>• Industrial Relations Guidelines, 2013<br>• Funding Guidelines, 2013	Total = 5<br><br>All 2013
Status Report	• Weekly status report<br>- Nov 2006<br>- Dec 2006-Jan 2007<br>- Feb 2007	Total = 3<br>2006 = I<br>2007= II
Service/Program Profile	• Service Provision map for Gurriny, no date<br>• Gurriny program profile, 2006<br>• Proposed service delivery framework, no date<br>• Workforce structure document, phases 1 & 3, 2010	Total = 4<br>By year<br>2006 = I<br>2010 = I<br>No date = II
Project proposal/funding requests	• Project proposal for transition officer position (x2), 2008<br>• Support letter for proposal, 2008<br>• Funding request, 2010<br>• Support letter from MP, 2008	Total = 5<br>By year<br>2008 = IIII<br>2010 = I
Readiness Assessment	• Readiness Assessment DRAFT GAP ANALYSIS – YARRABAH, 2013<br>• DRAFT READINESS ASSESSMENT (X2), 2012	Total = 3<br>2012 = II<br>2013 = I
OTHER	• Deed of Commitment, 2005<br>• Yarrabah budget table, 2012<br>• Service data doc, 2008	Total = 15<br><br>By year

	• Health indicators to measure, no date (approx. 2006-2008)<br>• Analyses of the sub-lease, 2017<br>• Analyses of Operating Deed (2017)<br>• Transition of Primary Health Care Services to Gurriny PRESENTATION, 2012<br>• Presentation - Community Controlled PHC In Cape York - Summary Of “Funds Pooling” Work-Stream: Yarrabah Supplement. December 2006<br><br>• Document about QH using Gurriny records, 2010 (fleshing out issues and options)<br>• QH document for QH staff on options re staff transition, 2014<br>• Transition Committee Terms of Reference, no date<br>• Operating Deed, 2016<br>• Gurriny Annual Report, 2017-2018<br>• Sub-Lease, 2009<br>• Transition position, 2010 (reporting on issues with QH)	2005 = I<br>2006 = I<br>2008 = I<br>2009 = I<br>2010 = II<br>2012 = II<br>2014 = I<br>2016 = I<br>2017 = III<br>No date = II
